# Determination of Regulated and Emerging Mycotoxins in Organic and Conventional Gluten-Free Flours by LC-MS/MS

**DOI:** 10.3390/toxins15020155

**Published:** 2023-02-14

**Authors:** Zoe Giannioti, Beatriz Albero, María Dolores Hernando, Luana Bontempo, Rosa Ana Pérez

**Affiliations:** 1 Traceability Unit, Research and Innovation Centre, Fondazione Edmund Mach, Via E. Mach 1, 38098 San Michele all’Adige, TN, Italy; 2 Centre for Agriculture, Food and Environment (C3A), University of Trento and Fondazione Edmund Mach Via E. Mach 1, 38098 San Michele all’Adige, TN, Italy; 3 Department of Environment and Agronomy, National Institute for Agricultural and Food Research and Technology (INIA), Spanish National Research Council (CSIC), Carretera de La Coruña Km 7.5, 28040 Madrid, Spain

**Keywords:** food safety, aflatoxins, *Fusarium*, rice flour, oat flour, matrix solid-phase dispersion, liquid chromatography–mass spectrometry

## Abstract

Gluten-free cereal products have grown in popularity in recent years as they are perceived as “healthier” alternatives and can be safely consumed by celiac patients, and people with gluten intolerance or wheat allergies. Molds that produce mycotoxins contaminate cereal crops, posing a threat to global food security. Maximum levels have been set for certain mycotoxins in cereal flours; however, little is known about the levels of emerging mycotoxins in these flours. The aim of this study was to develop an efficient, sensitive, and selective method for the detection of four emerging (beauvericin and enniatins A1, B, and B1) and three regulated (aflatoxin B1, zearalenone, and deoxynivalenol) mycotoxins in gluten-free flours. Ultrasound-assisted matrix solid-phase dispersion was used in the extraction of these mycotoxins from flour samples. The validated method was utilized for the LC-MS/MS analysis of conventional and organic wholegrain oat and rice flours. Six of the seven target mycotoxins were detected in these samples. Multi-mycotoxin contamination was found in all flour types, particularly in conventional wholegrain oat flour. Despite the low detection frequency in rice flour, one sample was found to contain zearalenone at a concentration of 83.2 μg/kg, which was higher than the level set by the European Commission for cereal flours. The emerging mycotoxins had the highest detection frequencies; enniatin B was present in 53% of the samples at a maximum concentration of 56 μg/kg, followed by enniatin B1 and beauvericin, which were detected in 46% of the samples, and at levels reaching 21 μg/kg and 10 μg/kg, respectively. These results highlight the need to improve the current knowledge and regulations on the presence of mycotoxins, particularly emerging ones, in gluten-free flours and cereal-based products.

## 1. Introduction

Gluten-free cereal products have consistently increased in popularity in recent years, not only because they are typically marketed as “healthier” alternatives, but also because they can be safely consumed by celiac patients, and people with gluten intolerance or wheat allergies. The global demand for rice flour by consumers has steadily increased, with a projected annual growth rate of 4.5% between 2022 and 2027 [1], while the demand for oat flour, another popular alternative to wheat flour, is forecast to increase by 5.5% annually between 2022 and 2029 [2].

Due to its hypoallergenic properties, rice flour is used in gluten-free products, such as pasta. It is also blended with wheat flour in bread-making and used in the production of biscuits, baby food, and waffle and pancake mixes [3]. Oats are a great source of protein and have been shown to possess several health benefits, including the maintenance of blood glucose levels and the reduction of oxidative stress [4]. Oat flour is often used in baby foods and ready-to-eat breakfast cereals [3], and trials are also being conducted for its use in bread production [5,6].

Mycotoxins are secondary metabolites produced by fungi that can be toxic to humans and animals [7]. Molds that produce mycotoxins contaminate cereal crops in the field pre-harvest through damage by insects or birds and harsh weather conditions; as well as post-harvest through improper storage, transport, and handling [8]. Mycotoxins are a threat to global food security, with the Food and Agriculture Organization of the United Nations (FAO) reporting the contamination of nearly 25% of the world’s food crops, with subsequent losses amounting to approximately one million tons per year [9]. Recent studies have actually reported much higher estimates, with mycotoxins detected in 60–80% of food products [10]. Cereals, including wheat, oats, maize, and rice, are among the crops that are most susceptible to mycotoxin contamination. This is a cause of great concern, given that they provide more than half of the calories humans consume each day [11,12].

The number of known mycotoxins exceeds 300, with the main ones being produced by the *Aspergillus*, *Fusarium*, and *Penicillium* species [11]. Aflatoxins (AFs), ochratoxin A (OTA), zearalenone (ZEN), deoxynivalenol (DON), and fumonisins (FBs) are the most important and prevalent mycotoxins found in food [11]. Given that their toxicological effects can be acute, these have been well researched for years, and several countries around the world have established legally acceptable levels in food. Based on EC Regulation 1881/2006, the maximum levels for AFB1 (2 μg/kg), DON (750 μg/kg), ZEN (75 μg/kg), and OTA (3 μg/kg) have been set for cereal flours [13].

In addition to the regulated mycotoxins, there is a group of currently non-regulated mycotoxins produced by *Fusarium* spp, also known as “emerging mycotoxins”, which includes beauvericin (BEA), enniatins (ENNs), and fusaproliferin (FUS) [14]. The main reason why no acceptable levels have been set for this group of mycotoxins is that there are not enough data regarding their occurrence, contamination levels, and toxicity [15].

In a review of studies on mycotoxins in cereals published between 2018 and 2020, it was concluded that 82% of these studies focused on AFB1, DON, ZEN, and OTA in maize, wheat, and rice, with the majority finding concentrations of AFB1 above the EU limit [16]. In the same review, it was mentioned that oat was the least investigated cereal, with only two relevant studies examining the content in DON, NIV, and T-2/HT-2 [17] and ZEN [18]. In a recent study on the presence of traditional mycotoxins in 200 wheat flour samples, DON and ZEN were the most prevalent, detected in 100% and 50% of the samples, respectively, at concentrations ranging between 53 and 2905 μg/kg for DON, and up to 50 μg/kg for ZEN [19]. DON and ZEN were similarly the most predominant mycotoxins among the 11 included in another study on wheat flours [20]; however, DON was the most frequently found mycotoxin among DON, ZEN, and their derivatives in both whole wheat and refined wheat flours [21]. Emerging mycotoxins in wheat flour samples have been less studied overall; however, one study suggested that enniatin A1 (ENNA1) was the most commonly detected mycotoxin (92.1%), followed by enniatin B (ENNB) (68.6%) and enniatin B1 (ENNB1) (39.2%) at lower concentrations [22]. 

The comparison of mycotoxin content in organic (labelled as organic, bio, or eco flour of any type) and conventional (labelled only with the type of cereal) flours, especially in gluten-free flours, is a topic that has scarcely been investigated to date. The primary debate around this comparison used to be whether organic cultivars would exhibit a higher mycotoxin content because no fungicides are allowed in these cultivation systems [23]. Several studies have demonstrated that fungicides effectively reduce the mycotoxin content of cereal cultivars [24], even up to as much as 90% [25]. However, a number of studies that examined the mycotoxin content of organic and conventional wheat flour drew different conclusions, with some concluding that there were no significant differences between the two types [26,27], and others finding that conventional flour had a higher mycotoxin content [28] or that organic samples were more contaminated [29]. To our knowledge, no studies have been conducted to evaluate the differences between organic and conventional gluten-free rice and oat flours.

The main objective of this study was to develop an efficient, sensitive, and selective analytical method for the detection of four emerging (ENNA1, ENNB, ENNB1, and BEA) and three regulated mycotoxins (DON, ZEN, and AFB1) in gluten-free flours. For this purpose, two different extraction methods, namely QuEChERS (which stands for “quick, easy, cheap, effective, rugged, and safe”) and ultrasound-assisted matrix solid-phase dispersion (UA-MSPD), were evaluated to select and optimize the method with the best performance. Twenty-eight samples, seven from each matrix (organic wholegrain oat flour, conventional wholegrain oat flour, organic rice flour, and conventional rice flour) were analyzed by LC-MS/MS using the validated extraction method.

## 2. Results and Discussion

### 2.1. Extraction Procedures

Three wholegrain oat flour and two rice flour samples were pre-screened using the QuEChERS extraction method to select one rice flour and one wholegrain oat flour without mycotoxins, or with the lowest mycotoxin content. These were then used in the recovery studies of both the QuEChERS and the UA-MSPD methods. The extracts after UA-MSPD were not concentrated in these first assays.

Recoveries using these methods were carried out by spiking the oat and rice flour blank samples with 100 μL of the standard mix containing between 0.4–40 μg/mL. Spiked blank-flour extracts were used for the quantification.

In preliminary assays using MSPD, lower than expected recoveries were obtained after cleanup. The lower post-cleanup recoveries could be attributable to the use of a non-acidified solvent (ACN:H_2_O 20:80, *v*/*v*) in the initial UA-MSPD method, since PSA alters the pH of the extract and is often linked to recovery losses of acidic components [30]. Extraction with acidic additives is recommended for pH-sensitive mycotoxins [31]. For these reasons, after the dSPE process, the extracts were diluted with ACN:H_2_O:formic acid (20:79:1, *v*/*v*/*v*) instead of the initial neutral mixture, with good results. To improve the detection limits, the extracts obtained after UA-MSPD were concentrated to 2 mL. Considering that the ratio of ACN:H_2_O present in the extracts would be approximately 1:1 (*v*/*v*) after the evaporation step, ACN:0.1% formic acid (1:1, *v*/*v*) was used for dilution (1:1) prior to chromatographic analysis.

**Figure 1:**
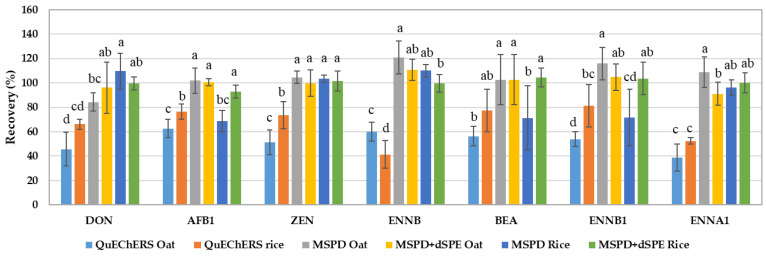
Recoveries obtained by QuEChERS and UA-MSPD extraction methods (with and without cleanup) on wholegrain oat and rice flours. Flours were spiked at 4–400 ng/mL (n = 3). Different letters indicate significant differences (LSD test, *p* < 0.05).

As shown in Figure 1, QuEChERS mean recoveries were significantly lower than UA-MSPD recoveries in the majority of cases, both with and without cleanup. Specifically, it was noted that UA-MSPD mean recoveries without cleanup yielded the highest values (ranging from 69.1% to 120.4%); QuEChERS mean recoveries ranged from 39% to 84%, and UA-MSPD mean recoveries with cleanup ranged from 91.1% to 104.7%. Given that, overall, the UA-MSPD method yielded higher recoveries for all the mycotoxins of interest while employing less extraction solvent and sample, this extraction method was optimized to improve the recoveries.

The temperature of the ultrasonic water bath and sonication time, two parameters that could affect the extraction, were evaluated to improve the UA-MSPD procedure’s extraction efficiency. Trials were carried out combining different sonication times (10 and 20 min) and temperatures (25 and 50 °C) to select the optimum extraction conditions. The possibility of eliminating the cleanup stage altogether was also assessed. However, the turbidity observed in the uncleaned extracts and the very high pre-cleanup recoveries, exceeding 150% for some compounds, indicated the presence of impurities, rendering this step necessary.

Overall, different conditions yielded the highest recoveries for every mycotoxin. Therefore, no clear pattern could be discerned when it came to the effect of sonication temperature and time on the extraction of all mycotoxins. The ranges were between 85–127% (25 °C–20 min), 91–115% (50 °C–20 min), and 84–111% (50 °C–10 min); however, the 50 °C–20 min conditions consistently yielded recoveries in the region of 100%, while significantly lower recoveries were obtained for ENNB and ENNA1 at 25 °C–20 min and 50 °C–10 min, respectively. Consequently, extraction at 50 °C and 20 min was deemed to be optimal and the methods were validated under these conditions.

### 2.2. Method Validation

The optimized extraction method was validated in terms of linearity accuracy, precision, and limits of quantification (LOQ). The linearity of the method was evaluated by injecting five spiked blank flour extracts with concentrations ranging from 0.25 to 5 ng/mL, equivalent to 1 to 20 µg/kg for the compounds with the lowest concentration in the mixture (ENNB, ENNB1, ENNA, BEA, and AFB1), while ZEN was present at a 10-fold concentration and DON at a 100-fold concentration. Good linearity was obtained with R^2^ ≥ 0.994 for all the compounds examined. Intra-day and inter-day precisions were evaluated to ascertain the method’s precision. Intra-day precision was calculated using the relative standard deviation (RSD) of three measurements for each concentration on the same day, while inter-day precision was calculated using the RSD of measurements taken two weeks apart. The results showed an overall intra-day RSD% in the range of 3–12% and an inter-day RSD% of 6–13% (Table 1), both of which were within the accepted variable limits. Sensitivity was assessed by the LOQ, which was considered to be the lowest spiking level (Table 1).

The method’s accuracy was evaluated by obtaining the recoveries of the target analytes from the flour samples at two different concentration levels. The low level included ENNA1, ENNB1, ENNB, BEA, and AFB1 at 1 μg/kg, ZEN at 10 μg/kg, and DON at 100 μg/kg (limits of quantification, LOQs), while the high level included ENNA1, ENNA, ENNB, BEA, and AFB1 at 20 μg/kg, ZEN at 200 μg/kg, and DON at 2000 μg/kg (20 times LOQs). As shown in Table 1, the recoveries obtained were in the range of 87–122% in wholegrain oat flour and 85–122% in rice flour.

Table 2 depicts a comparison between this method and those reported by other authors for the determination of mycotoxins in cereal samples. Taking the reported studies into account, the devised method allowed for the analysis of these mycotoxins with similar or higher recoveries, employing lower extraction solvent volumes and inexpensive column cleanup. In most cases, the sample size was 5 g and the volume of extraction solvents used was in the range of 10–35 mL [19,32,33]. The SLE followed by the IACs summarized in Table 2 often required large amounts of extraction solvents, such as 25 mL of ACN:H_2_O:formic acid (79:20:1, *v*/*v*) [34], 30 mL of pure MeOH [22]; or 25 mL of dichloromethane [27], 100 mL of MeOH/H_2_O (80:20, *v*/*v*) [35]; or 100 mL of ACN:H_2_O (90:10%, *v*/*v*) [36]. Larger sample sizes of up to 10 g were also commonly employed in the extraction of IACs [35]. The high cost associated with IACs is another factor that has limited their use in the cleanup phase [37].

The LOQ values were also comparable to those in the other studies, with 1 µg/kg for ENNs and BEA; and 10 and 100 µg/kg for ZEN and DON, respectively. The limits obtained for ENNs in this study were similar to those recently reported in the analysis of 20 mycotoxins in wheat flour by solid–liquid extraction, although they obtained lower LOQs for ZEN, DON, and AFB1 [34]. In another study, the limits reported for ENNs and BEA in wheat flours by SPE were similar or higher than those obtained using the MSPD method reported in this investigation [33]. It is not possible to draw a general conclusion on the limits obtained for AFB1 and ZEN in oat and rice flours in comparison to those obtained by other authors (summarized in Table 2), as the reported values were higher, similar, or lower than those obtained in our research. Although the LOQs achieved in our study for DON (100 µg/kg) were higher than those reported in wheat flours with different extraction methods (Table 2), higher concentrations of this mycotoxin (43, 50, 60, and 75 µg/kg) were found in wheat and maize flours [19,21,32,38].

The method predominantly used for the analysis of mycotoxins in flours was solid–liquid extraction (SLE), followed by one or more immunoaffinity columns [21,35,36,39] (see Table 2). This method generally yielded good recoveries of the mycotoxins DON, ZEN, and AFB1 in wheat flour. The QuEChERS method has also been reported for the extraction of mycotoxins in wheat flour [19] and corn flour [32], including the analysis of AFB1, DON, and ZEN with good recoveries. An MSPD method for multi-mycotoxin determination in different flours has been reported with the use of C18 as the dispersive sorbent and 20 mL of acetonitrile/methanol (50/50 *v*/*v*) and 1 mM ammonium formate as the extraction solvent [40]. In this investigation of 14 target mycotoxins in different cereal flours, 9 were detected solely in wheat flour samples, whereas BEA was present in a high percentage (23%) of samples and ZEN in a low percentage (5%) [40]. The mycotoxins AFB1, DON, ZEN, and BEA were included in other studies’ evaluation, but not the emerging ENNs. The authors noted that BEA has been less investigated than other mycotoxins and that their findings demonstrated the need for future in-depth research into this mycotoxin. It is for this reason that this mycotoxin was included in our study. As discussed below in Section 2.3, BEA was detected in 46% of the analyzed flour samples, primarily in oat flours, with levels as high as 10 µg/kg.

Our analysis included the mycotoxins DON, ZEN, BEA, and AFB1 because they are frequently detected in cereal flours (Table 2). On the other hand, no data are available on the occurrence of ENNA1; however, ENNB and ENNB1 extracted from wheat flours by SLE have been detected in small percentages [33,34] (Table 2). 

In this study, the UA-MSPD method was validated for the analysis of AFB1, DON, ZEN, BEA, ENNB, ENNB1, and ENNA1 in oat and rice flours, and subsequently applied to commercial samples.

### 2.3. Analysis of Mycotoxins in Flour Samples

Once the analytical method was optimized and validated, it was applied to the comparative analysis of target mycotoxins in conventional and organic gluten-free flours. The standard addition method was used to avoid matrix effects, and achieve adequate quantification of mycotoxins in oat and rice flours. Table 3 summarizes the levels of mycotoxins detected in the samples analyzed. Six of the seven target mycotoxins were detected, while two (AFB1 and ZEN) were only found in one of the conventional rice samples analyzed. With the exception of ENNA1, which was not found in the conventional rice flours, all the emerging mycotoxins were detected in all the flour types. Quantity and frequency were dependent on the type of sample. The levels and detection frequencies of these mycotoxins were higher in oat flours, particularly conventional flours, than in rice flours. Thus, the four target emerging mycotoxins were found in all the conventional wholegrain oat flours evaluated, although their frequency and quantity were lower in organic flours. In contrast, their presence was low in both conventional and organic rice flours, with only 14% showing positive results at levels < LOQ (see Table 3). Except for the presence of AFB1 (>LOQ) and ZEN (83.2 µg/kg) in conventional rice samples, there were few differences in the content of these emerging mycotoxins between conventional and organic rice samples. Despite the low levels of mycotoxins in rice, the ZEN level found was higher than the level established by the European Commission for cereal flours. High ZEN levels in certain corn flours have been described in the literature [32]. BEA was detected in all the conventional wholegrain oat flours and in 57% of organic wholegrain oat flours; however, its presence was low in conventional and organic rice flours, with 14% positive results at levels < LOQ.

Although there is limited information regarding the presence of emerging mycotoxins in flours and the studies are usually focused on wheat flours (see Table 3), their presence in a variety of cereals on the Spanish market was investigated by Meca et al. [41]. In this study, ENNs had the highest contamination frequency (73%) compared to BEA (33%) and FUS (8%). Specifically, high levels of ENNA1 (338 mg/kg) were found in one oat flour sample and BEA was detected at a concentration of 4 mg/kg. Oueslati et al. [22] reported that in cereals and derived products, ENNA1 was the most commonly detected mycotoxin (92.1%) at levels ranging from 11.1 to 480 mg/kg, followed by ENNB (68.6%) and ENNB1 (39.2%) at lower concentrations. A more recent study [42] found that 9 of the 20 mycotoxins examined were present in pasta samples consumed in Morocco; ENNB and ENNB1 were the predominant mycotoxins, followed by ZEN and DON, whereas AFB1 was detected in only 2 samples.

The analysis of a total of 114 samples of wheat, rye, oat, rice, and corn flours, among others, reported a 33.3% incidence of AFB1 in rice flours [39]. Contamination of wheat, corn, rice, and oat flours with traditional mycotoxins and BEA was also reported in an earlier study [40]. This study concluded that BEA was the most prevalent mycotoxin in wheat, rice, and oat flour, occurring in 6/25, 3/3, and 2/3 samples, respectively, with total concentrations ranging from 150 to 720 μg/kg [40].

Even though AFB1 and DON were among the main mycotoxins evaluated in the flours, with a focus on wheat flours and products, no levels of these mycotoxins were found in studies involving a large number of samples (15, 40, 50, or 200 samples in the studies [19,20,34,40]). Nevertheless, data on the presence of AFB1 in a total of 114 samples of different types of flour marketed in Serbia have been reported [39]. The flours examined were mainly corn and wheat flours (with 56 and 20 samples, respectively), along with 6 rice flours. AFB1 results revealed that this mycotoxin was present in 48.3% of the corn flours (27 samples) and 33.3% of the rice flours, but not in the wheat flours (20 samples). Moreover, Noroozi et al. [35] found that all the flour samples (n = 5) were contaminated with AFB1 at levels in the range from 0.5 to 3.4 µg/kg. In another study, the presence of AFB1 was evaluated in conventional and organic flour samples, and it was only found in corn flour samples (26%), revealing that the EU limit (2 mg/kg) was exceeded in one case [27]. No significant differences in AFB1 contamination were observed between the two categories. In our analysis, none of the samples contained detectable levels of DON, and only one conventional rice sample had detectable levels of AFB1 along with a ZEN concentration that is above the maximum limit established by the European Commission for this mycotoxin. As discussed, ENNs and BEA were found in all the analyzed flour types, with the ranges and detection frequencies listed in Table 3. 

Despite the high number of samples found to contain mycotoxins, they were all below the tolerable levels established by the European Commission, with the exception of ZEN. Our findings indicate that storage conditions and post-harvest handling should be given greater consideration to minimize mycotoxin levels.

## 3. Conclusions

A rapid, selective, and efficient method based on UA-MSPD and dSPE cleanup was successfully developed to analyze seven mycotoxins (three regulated and four emerging) in gluten-free flours. This method allowed the analysis of these mycotoxins with similar or higher recoveries compared to other methods, employing lower extraction solvent volumes and an inexpensive cleanup. The recovery values were satisfactory (85–122%) and the LOQs were between 1 and 100 µg/kg. Following the method validation, the procedure was applied to analyze conventional and organic oat and rice flours, resulting in six of the target mycotoxins being detected in the samples. ENNB, ENNB1, ENNA1, and BEA were detected in all the conventional oat flours and in a high percentage of organic oat flour, whereas low detection frequencies were found in conventional and organic rice flours. These results reveal the presence of multi-mycotoxin contamination and highlight the need for more data on the incidence and toxicity of mycotoxins in these cereal flours, particularly of the emerging ones. Furthermore, the detection of ZEN at a level higher than permitted by EU regulations is concerning for food quality and safety. Our findings underline the need for extensive quality controls and anti-contamination measures throughout the food production and supply chain processes.

## 4. Materials and Methods

### 4.1. Chemicals and Reagents

ENNA1, ENNB1, and ENNB standards were purchased from Sigma-Aldrich (Steinheim, Germany); DON and AFB1 standards from LGC (Teddington, UK); and BEA and ZEN standards from Dr. Ehrenstorfer (Augsburg, Germany). Individual stock solutions were prepared at 100 μg/mL in dimethylsulfoxide (DMSO), except for BEA, which was prepared at 100 µg/mL in acetonitrile (ACN) and DON at 500 µg/mL in methanol (MeOH). Working standard solutions were prepared by appropriately diluting the stock standard solutions with ACN and stored in amber vials at −20 °C. HPLC-grade ACN and MeOH were purchased from Sigma-Aldrich (Steinheim, Germany). Ammonium formate, 99% purity, and NaCl were purchased from Sigma-Aldrich (Steinheim, Germany). Formic acid and acetic acid were acquired from Honeywell Fluka (Seelze, Germany). Bulk Extrabond^®^ C18, PSA, and MgSO_4_ were obtained from Scharlab (Barcelona, Spain). Ultrahigh-purity water (H_2_O) was obtained from a MilliQ water purification system (Millipore, Madrid, Spain).

### 4.2. Sampling

The applicability of the method was tested on 28 flour samples (7 organic wholegrain oat, 7 conventional wholegrain oat, 7 organic rice, and 7 conventional rice) purchased from Spanish supermarkets and online retailers. 

### 4.3. Sample Preparation

Based on the QuEChERS method reported by Tolosa et al. [43] and the UA-MSPD extraction reported by Albero et al. [44], two methods were tested for the simultaneous extraction of the 7 mycotoxins, both with some modifications. 

#### 4.3.1. QuEChERS

A 1 g sample of homogenized flour was placed in a 15 mL polypropylene tube and mixed with 5 mL of water containing 2% formic acid. The extraction solvent was added (5 mL of ACN) after 30 min and taken to a horizontal shaker for mixing (10 min at 2000 rpm). Thereafter, 2 g of MgSO_4_ and 0.5 g NaCl were added, and the mixture was immediately shaken for 5 min to form a uniform mix; and then, centrifuged for 5 min at 5000 rpm and 20 °C. The organic phase was isolated, and 1.5 mL was purified by dispersive solid-phase extraction (dSPE) with 0.1 g of C18 and 0.3 g of MgSO_4_, vortexing the mixture for 1 min and centrifuging for 5 min (10,000 rpm). The purified extract was finally diluted (1:1, *v*/*v*) with formic acid at 0.1% and filtered through a 0.2 μm nylon filter prior to chromatographic analysis.

In the recovery studies, the samples were spiked and allowed to sit for 1 h before the extraction procedure was carried out.

#### 4.3.2. UA-MSPD with dSPE Cleanup

In a glass mortar, 1 g of flour along with 1 g of sand and 0.5 g of C18 were thoroughly mixed with a pestle and then, placed in a 20 mL glass column with cellulose frits at the bottom and closed with a stopcock. The extraction was carried out twice using 3.5 mL of ACN:H_2_O:acetic acid (79:20:1, *v*/*v*/*v*) in an ultrasonic water bath (360 W, 50–60 Hz) at 50 °C for 20 min. The extracts were collected in 15 mL polypropylene tubes in a vacuum manifold and then, concentrated to 2 mL. For the cleanup by dSPE, an aliquot (1 mL) was placed in a 2 mL Eppendorf tube containing 50 mg PSA, vortexed for 2 min, and centrifuged (5 min, 10,000 rpm). Finally, a 0.5 mL aliquot of the purified extract was diluted (1:1 *v*/*v*) with ACN:0.1% formic acid (50:50, *v*/*v*) and filtered through a 0.2 μm nylon filter prior to analysis.

Similar to the QuEChERS method, samples in the recovery studies were spiked and allowed to sit for 1 h before proceeding with the extraction.

### 4.4. LC-MS/MS Analysis

Analyses were performed on an Agilent 1200 LC system (Waldbronn, Germany). A Kinetex^®^ XB-C18 (100 × 3 mm i.d., 2.6 μm particle size) LC column with a C18 security guard cartridge from Phenomenex (Torrance, CA, USA) was used. Chromatographic separation was performed at a flow rate of 0.3 mL/min with the column temperature set at 30 °C. The mobile phase was a time-programmed gradient using H_2_O (eluent A) and MeOH (eluent B), both containing 3 mM ammonium formate and 0.1% (*v*/*v*) formic acid. Gradient elution started isocratically at 90% A for 1 min. B was then increased linearly to 100% within 5 min and kept constant for 4 min. Finally, B was decreased to 10% in 6 min and equilibrated for 10 min.

Mass spectrometry was performed with an Agilent 6420 triple-quadrupole mass spectrometer (Waldbronn, Germany) equipped with an electrospray ionization interface, operating in positive and negative ion modes. The following mass spectrometer parameters were set: drying gas temperature of 300 °C, drying gas flow rate of 9 L/min, nebulizer gas pressure of 35 psi, and capillary voltage of 3500 V.

Multiple reaction monitoring (MRM) mode was applied for the identification and quantification of the analytes. As shown in Table 4, one precursor and two product ions were selected for each mycotoxin, along with their optimal collision energies and fragmentor voltages. The analytes were identified based on their retention times and assessment of their quantifier and qualifier transitions. Specifically for their confirmation, the retention time of each mycotoxin had to be within ±0.2 min of the expected time, while the qualifier-to-quantifier ratios had to be at least 20%.

### 4.5. Statistical Analysis

Data were statistically analyzed using STATGRAPHICS software (version XVII). Significant differences between treatments were determined by one-way analysis of variance (ANOVA) with a Fisher’s least significant difference procedure (LSD, *p* < 0.05).

## Figures and Tables

**Table 1:** Recovery results (mean ± RSD, %) (n = 3), inter-day precision, and limits of quantification (LOQ) in wholegrain oat and rice flours.

	Wholegrain Oat Flour				Rice Flour				LOQ (μg/kg)
	Recovery	Recovery	Inter-Day RSD% *		Recovery	Recovery	Inter-Day RSD% *		
	1–100 μg/kg	20–2000 μg/kg			1–100 μg/kg	20–2000 μg/kg			
DON	105.4 ± 11.5	110.1 ± 8.0	11.7		102.9 ± 5.0	99.6 ± 5.5	6.9		100.0
AFB1	98.5 ± 9.6	110.3 ± 3.7	12.1		84.8 ± 7.8	92.9 ± 5.2	8.4		1.0
ZEN	99.4 ± 6.7	113.5 ± 7.8	13.3		100.4 ± 7.8	101.7 ±8.2	11.8		10.0
ENNB	108.3 ± 9.0	94.5 ± 8.2	11.6		97.7 ± 5.4	99.8 ± 7.2	6.0		1.0
BEA	121.7 ± 11.5	96.5 ± 7.3	9.4		121.6 ± 7.6	104.4 ± 7.7	8.7		1.0
ENNB1	87.4 ± 6.9	99.0 ± 2.6	11.4		107.7 ± 8.6	103.6 ± 6.6	5.7		1.0
ENNA1	98.9 ± 6.2	90.6 ± 8.9	11.5		89.8 ± 5.0	100.3 ± 8.2	7.5		1.0

* RSD: relative standard deviation.

**Table 2:** Summary of studies in which target mycotoxins were detected in cereal flours, including recovery values and LOQs.

Flour Type(s)	Analytes (Common with This Study)	Method	Recoveries in Flours %	LOQs	Mean Levels, μg/kg (Positive/Total Samples)	Ref.
Wheat	20 mycotoxins (AFB1, DON, ZEN, ENNB, ENNA1)	SLE shaking	73–100	0.1–3	ENNB; 0.6 (2/54); ENNB1 9.5 (2/54); DON 79 (49/54)	[34]
Wheat	1 mycotoxin (AFB1)	SLE + IAC	96–99	0.15	AFB1 0.5–3.4 (5/5)	[35]
Corn and wheat	3 mycotoxins (DON)	SLE shaking + IAC	86–102	43–47	DON < LOQ–853 (73/104)	[38]
Wheat	12 mycotoxins (AFB1, DON, ZEN)	QuEChERS	75–116	1–50	DON 53–2905 (200/200); ZEN < LOQ (102/200)	[19]
Wheat	9 mycotoxins (DON, ZEN)	SLE shaking + IAC	82–99	5–60	DON < LOD–924.6 (66/85); ZEN < LOD–17 (6/85)	[21]
Wheat, buckwheat, rye, oat, barley, rice, millet, and corn	5 mycotoxins (AFB1)	SLE shaking + IAC	53–85	0.1	AFB1 0.53–4.76 (28/66)	[39]
Wheat	5 mycotoxins (ENNA1, ENNB, ENNB1, BEA)	SLE shaking	61–127	1–4	ENNB 9.8 (1/4); ENNB1 2.3 (1/4)	[33]
Corn	6 mycotoxins (AFB1, DON, ZEN)	QuEChERS	97–116	2–75	AFB1 < LOQ–1060 (25/40) ZEN < LOQ–3170 (14/40)	[32]
Wheat and corn flours	1 mycotoxin (AFB1)	UAE	78–100	0.50	0.2–3.7 (9/42)	[27]
Wheat	11 mycotoxins (AFB1, DON, ZEN)	SLE shaking	72–115	1.0–2.3	DON 17.5–976 (13/15); ZEN 1.9–21.1 (5/15)	[20]
Wheat, corn, rice, soy, oat, and multi-cereal	14 mycotoxins (AFB1, DON, ZEN, BEA)	MSPD	73–89	1–31	DON 45–367 (6/40); ZEN 39–70 (2/40); BEA 150–720 (9/40)	[40]
Rice	5 mycotoxins (AFB1)	SLE and IAC	88–90	0.20	LOD-9.8 (11/37)	[36]
Wholegrain oat and rice	7 mycotoxins (AFB1, DON, ZEN, ENNB, ENNB1, ENNA1, BEA)	MSPD and dSPE	85–122	1–100	AFB1 < LOQ (1/28); ZEN 83 (1/28); ENNA1 < LOQ-7.1 (12/28); ENNB < LOQ-56 (15/28); ENNB1 < LOQ-21 (13/28); BEA < LOQ-10 (13/28)	Present work

**Table 3:** Mycotoxin concentrations in investigated samples of flour.

Type and Number (% Frequency)	Levels in µg/kg (Frequency of Detection)					
	AFB1	ZEN	ENNB	BEA	ENNB1	ENNA1
Conventional wholegrain oat	-	-	3–56	<LOQ-10	2–21	<LOQ-2
n = 7 (100%)	(0%)	(0%)	(100%)	(100%)	(100%)	(100%)
Organic wholegrain oat	-	-	<LOQ-6.7	<LOQ-1.1	1.4–10	<LOQ-7.1
n = 7 (71%)	(0%)	(0%)	(71)%	(57%)	(57%)	(57%)
Conventional	<LOQ	83.2	<LOQ	2.2	<LOQ	-
rice n = 7 (29%)	(14%)	(14%)	(14%)	(14%)	(14%)	(0%)
Organic rice	-	-	<LOQ	<LOQ	<LOQ	<LOQ
n = 7 (29%)	(0%)	(0%)	(29%)	(14%)	(14%)	(14%)

**Table 4:** Optimized MRM conditions for the analysis of the mycotoxins of interest in this study.

Compound	MRM 1	CE (eV)	Fragmentor (V)	MRM 2	CE (eV)	Fragmentor (V)	Polarity
AFB1	313 > 128.1	70	165	313 > 285.2	20	100	Positive
DON	297.1 > 249.1	10	100	297.1 > 77	80	100	Positive
ZEN	317 > 175	20	195	317 > 131	28	195	Negative
BEA	801.5 > 262	32	180	801.5 > 244	36	180	Positive
ENNA1	685 > 228	32	150	685 > 210	32	150	Positive
ENNB1	672 > 196	32	170	671.4 > 214	60	170	Positive
ENNB	657 > 196	32	160	657 > 214	32	160	Positive

## Data Availability

Data are contained within the article.
